# Vitamin D deficiency as a risk factor for infection, sepsis and mortality in the critically ill: systematic review and meta-analysis

**DOI:** 10.1186/s13054-014-0660-4

**Published:** 2014-12-05

**Authors:** Kim de Haan, AB Johan Groeneveld, Hilde RH de Geus, Mohamud Egal, Ard Struijs

**Affiliations:** Department of Intensive Care, Erasmus Medical Centre, Mailbox 2040, H603a, 3000CA Rotterdam, The Netherlands; Department of Intensive Care, Erasmus Medical Centre, Mailbox 2040, H603, 3000CA Rotterdam, The Netherlands; Department of Intensive Care, Erasmus Medical Centre, Mailbox 2040, H619, 3000CA Rotterdam, The Netherlands

## Abstract

**Introduction:**

In Europe, vitamin D deficiency is highly prevalent varying between 40% and 60% in the healthy general adult population. The consequences of vitamin D deficiency for sepsis and outcome in critically ill patients remain controversial. We therefore systematically reviewed observational cohort studies on vitamin D deficiency in the intensive care unit.

**Methods:**

Fourteen observational reports published from January 2000 to March 2014, retrieved from Pubmed and Embase, involving 9,715 critically ill patients and serum 25-hydroxyvitamin D_3_ (25 (OH)-D) concentrations, were meta-analysed.

**Results:**

Levels of 25 (OH)-D less than 50 nmol/L were associated with increased rates of infection (risk ratio (RR) 1.49, 95% (confidence interval (CI) 1.12 to 1.99), *P* = 0.007), sepsis (RR 1.46, 95% (CI 1.27 to 1.68), *P* <0.001), 30-day mortality (RR 1.42, 95% (CI 1.00 to 2.02), *P* = 0.05), and in-hospital mortality (RR 1.79, 95% (CI 1.49 to 2.16), *P* <0.001). In a subgroup analysis of adjusted data including vitamin D deficiency as a risk factor for 30-day mortality the pooled RR was 1.76 (95% CI 1.37 to 2.26, *P* <0.001).

**Conclusions:**

This meta-analysis suggests that vitamin D deficiency increases susceptibility for severe infections and mortality of the critically ill.

**Electronic supplementary material:**

The online version of this article (doi:10.1186/s13054-014-0660-4) contains supplementary material, which is available to authorized users.

## Introduction

Vitamin D deficiency, defined as serum 25-hydroxyvitamin D_3_ (25 (OH)-D) concentrations below 50 nmol/L, is highly prevalent in Dutch critically ill patients [1]. Several studies in critically ill patients report associations between vitamin D deficiency, a disturbed parathyroid hormone (PTH)-vitamin D axis and increased mortality [2-5]. A biological basis how hypovitaminosis D may cause mortality could be hypocalcaemia. Hypocalcaemia is a well-known abnormality in critically ill patients in the course of sepsis and rhabdomyolysis [6]. Second, vitamin D regulates both innate and adaptive immune systems. Vitamin D deficiency leads to immune dysregulation and has been proposed as an underlying pathogenic mechanism of infections [7]. Third, vitamin D deficiency is associated with increased markers of systemic inflammation associated with multi-organ failure [8]. Moreover hypovitaminosis D reduces, despite maximal upregulation of PTH levels, formation of 1,25-dihydroxyvitamin D_3_ (1,25 (OH)-D) at the tissue level. This may be critical in mediating the beneficial pleiotropic functions of vitamin D, involving innate immunity, mucosal barrier and endothelial function. Recently, a systematic review and meta-analysis including observational and interventional studies on vitamin D in non-critically ill patients, suggests an association of deficiency with cardiovascular diseases, diabetes, and all-cause mortality in the former but not in the latter studies [9]. In non-critically ill patients, of a prior meta-analysis of 18 randomised controlled trials, intake of supplementary doses of vitamin D was associated with a 7% decrease in mortality [10].

Therefore, we conducted a systematic review to pool the available data and to study the possible effect of vitamin D deficiency in critically ill patients on the incidence of infection, sepsis and association with mortality.

## Materials and methods

### Search strategy

The report of this protocol-driven systematic review and meta-analysis follows the Preferred Reporting Items for Systematic reviews and Meta-Analyses (PRISMA) and Meta-analysis Of Observational Studies in Epidemiology guidelines (MOOSE) [11]. A Medline, Embase and Cochrane Library search was conducted with the help of biomedical information specialists, limited to publications from January 2000 until March 2014 in humans. The search consisted of two terms: vitamin D and intensive care. The controlled thesaurus terms we used for vitamin D were vitamin D and vitamin D deficiency. The concept intensive care was covered by the following keywords: intensive care unit (ICU), intensive care nursing, critical care and critically ill patients. For Medical Subject Heading (MeSH) terms and search strategy see S1 Table 1 in Additional file 1. References of included articles were cross-checked for other relevant studies. One author [12] was successfully contacted twice because not all required information could be retrieved from the publication.

### Study selection

Two independent authors, a researcher (KdH) and an intensivist (AS) screened titles and abstracts for eligibility. In case of disagreement, agreement of a third author (ME) provided a final option. Studies eligible for inclusion in the systematic review were observational studies describing ICU patients, reporting on serum 25 (OH)-D concentrations, outcomes, and those written in English. Studies in which participants were younger than 18 years, sample size was less than 20 patients and participants suffered from parathyroid disease, human immunodeficiency virus infection and end-stage renal disease requiring chronic dialysis were excluded. Abstracts, letters, reviews and conference articles were excluded as well.

### Definitions

In this meta-analysis vitamin D deficiency was *a priori* defined as a reported serum concentration <50 nmol/L, as advocated by the American Institute of Medicine [13]. However, the included studies unfortunately reported a wide range of cutoffs for vitamin D deficiency. Prior to analysing the data for each study, agreement was negotiated between two authors, KdH and AS, which vitamin D cutoffs to be used in the meta-analysis, see Table 1. Due to the designed search strategy, there was no clear definition of infection and sepsis between included studies. The reported occurrence of infections comprised any kind of infections such as pneumonia, urinary tract, bacteraemia and intra-abdominal infections. The included studies defined sepsis varying from having positive blood cultures or systemic inflammatory response syndrome (SIRS) criteria combined with a source of infection. We reported the complete overview of definitions used in S2 Table 2 in Additional file 1. Mortality rates were extracted as in-hospital and 30-day mortality. Thirty-day mortality rates are well described and defined in the included articles. All other definitions of mortality, like ICU mortality, in-hospital mortality, acute in-hospital mortality, were taken together as in-hospital mortality. When articles separately reported ICU and in-hospital mortality, we combined numbers together in the analysis as in-hospital mortality.

**Table 1:** **Overview of publications used in the meta-analysis**

**Author**	**Year of publication**	**Study population**	**Study design**	**Number of patients**	**Endpoints**	**Comparison** ***(in meta-analysis)***	**Study** ^*^ **quality**
Amrein *et al.* [25]	2014	Medical, surgical ICU	Retrospective, cohort	655	Sepsis, in-hospital mortality	<50 nmol/l vs >75 nmol/l	7
Arnson *et al.* [21]	2012	Medical, surgical ICU	Prospective, cohort	130	Infections	≤50 nmol/l vs >50 nmol/l	6
Aygencel *et al.* [24]	2013	Medical ICU	Prospective, cohort	201	Infections, sepsis, in-hospital mortality	<50 nmol/l vs ≥50 nmol/l	4
Braun *et al.* [5]	2012	Medical, surgical ICU	Two-centre, retrospective, cohort	1,325	Sepsis, 30-day, in-hospital mortality	≤37 nmol/l vs ≥75 nmol/l	8
Braun *et al.* [3]	2011	Surgical ICU	Two-centre, retrospective, cohort	2,399	Infections, 30-day, in-hospital mortality	≤37 nmol/l vs ≥75 nmol/l	8
Flynn *et al.* [22]	2012	Medical, surgical ICU	Prospective, cohort	66	Infections, sepsis, in-hospital mortality	≤50 nmol/l vs >50 nmol/l	2
Higgins *et al.* [23]	2012	Medical, surgical ICU	Prospective, cohort	196	Infections, sepsis, 30-day mortality	≤30 nmol/l vs ≥60 nmol/l	7
Lucidarme *et al.* [12]	2012	Medical, surgical ICU	Prospective, cohort	134	30-day mortality	>15- ≤30 vs ≥60 nmol/l	5
Matthews *et al.* [30]	2012	Medical ICU	Prospective, cohort	258	In-hospital mortality	≥10- ≤32 vs 67-97 nmol/l	3
Moromizato *et al.* [26]	2014	Medical, surgical ICU	Two-centre, retrospective, cohort	3,386	Sepsis	≤37 nmol/l vs ≥75 nmol/l	8
Nair *et al.* [27]	2012	Medical ICU	Prospective, cohort	100	30-day-, in-hospital mortality	<25 nmol/l vs ≥50 nmol/l	6
Remmelts *et al.* [28]	2012	Ward, medical ICU	Prospective, cohort	272	30-day mortality	≤50 nmol/l vs ≥75 nmol/l	7
Su *et al.* [29]	2013	Medical, surgical ICU	Prospective, cohort	156	30-day mortality	≤37 nmol/l vs ≥75 nmol/l	6
Venkatram *et al.* [4]	2011	Medical ICU	Retrospective, cohort	437	Sepsis, in-hospital mortality	≤50 nmol/l vs ≥75 nmol/l	4

^*^Study quality assessed by the Newcastle-Ottawa scale, see S8 in Additional file 1. ICU, intensive care unit.

### Statistical analysis

Review Manager (version 5.2 for Windows, The Cochrane Collaboration, 2011) was used for the analysis. Occurrence of infections, sepsis and mortality, as defined in the studies, was calculated for each individual study and the estimated risk ratios (RRs) were pooled comparing the effect of deficient levels (sufficient levels as reference) of 25 (OH)-D with the use of the inverse variance (IV) method in a random-effects model, yielding RRs and 95% confidence intervals (CIs). The IV method was used because of the assumption that less variance in a study should contribute to its weight in significance. A random-effects model was used due to expected heterogeneity between studies. If raw data was not available to calculate the RRs, we used the reported odds ratios (ORs) and converted the reported ORs to RRs with corresponding 95% CIs. Otherwise, we manually calculated the RRs from the available data. Additionally, taking confounding into account, we decided to perform a subgroup analysis of adjusted data reported on 30-day mortality. Unfortunately, most studies do not report adjusted data on infections, sepsis and in-hospital mortality, so we were not able to make a subgroup analysis of those. Subgroup analyses were performed to examine the difference per outcome based on study design. To determine publication bias we used funnel plots (Figures S3-S7 in Additional file 1). Heterogeneity was assessed with the use of the Cochran Q statistics and the I^2^ test. We used the Newcastle-Ottawa scale to evaluate the quality of included studies. This scale uses a star system (with a maximum of nine stars) to evaluate a study in three domains: selection of participants, comparability of study groups, and the ascertainment of outcomes of interest. We judged studies that received a score of nine or eight stars to be at low risk of bias, studies that scored seven or six stars to be at medium risk, and scores below six to be at high risk of bias (S8a,b in Additional file 1).

## Results

### Search strategy

**Figure 1:**
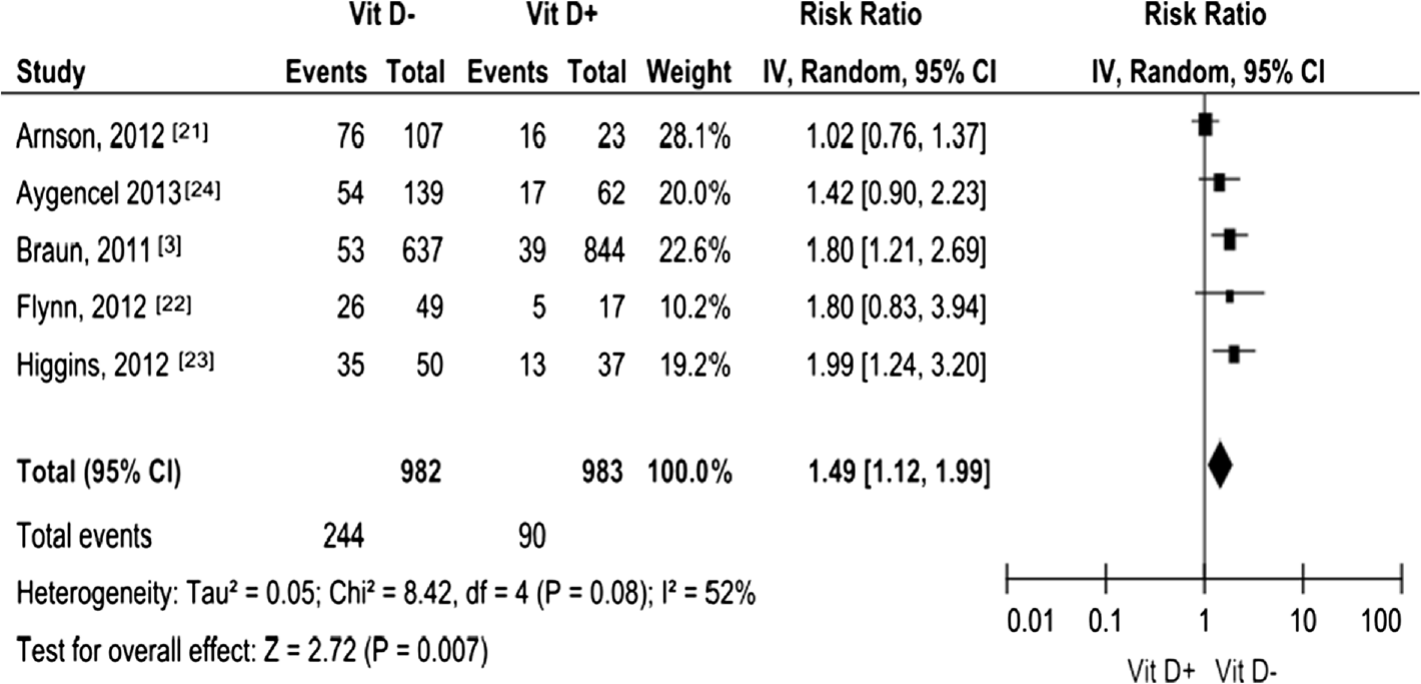
**Forest plot of studies comparing deficient vitamin D levels with sufficient vitamin D levels on infection.** CI, confidence interval; IV, inverse variance; Vit D-, deficient vitamin D level; Vit D+, sufficient vitamin D level.

A total of 381 studies were screened; 358 were excluded for the following reasons: irrelevant (n = 229), review (n = 7), study design or too small in sample size (n = 26), only focused on vitamin D metabolism (n = 45), pediatric studies (n = 46) and animal studies (n = 5), after detailed evaluation, one additional study was excluded because of duplicates. All studies included in this analysis (n = 14) were prospective cohort studies (n = 9) or had retrospective designs (n = 5). We were not able to include all studies because they described different outcomes like effect on delirium, anti-microbial peptide levels and 90-day mortality [8,14,15] or their results [16] were not formatted to allow combining the results with the other studies. We also excluded four interventional trials because they were small in sample size and none reported on infections, sepsis and mortality [17-20], (S9 Figure 1 in Additional file 1).

### Study characteristics

Fourteen observational reports published from January 2000 until March 2014 involving 9,715 critically ill patients were included. The average serum 25 (OH)-D level of the study population was 45 nmol/L. Mean age was 62 years and the majority of the patients, 53%, were male. The presence of infections was 31% and sepsis occurred in 28% of the patients. The average 30-day mortality in this study cohort was 17.5% and the in-hospital mortality rate 18.4%. Characteristics of the studies are presented in Table 1. For a complete overview of study characteristics of studies included in the meta-analysis see S2 Table 2 in Additional file 1.

### 25 (OH)-D levels and infections

Five [3,21-24] of the fourteen studies reported on infections. We pooled the manually calculated RRs for the effect of low levels of 25 (OH)-D on infections (Figure 1), including 1,967 patients. The pooled RR was 1.49 (95% CI 1.12 to 1.99).

### 25 (OH)-D levels and sepsis

**Figure 2:**
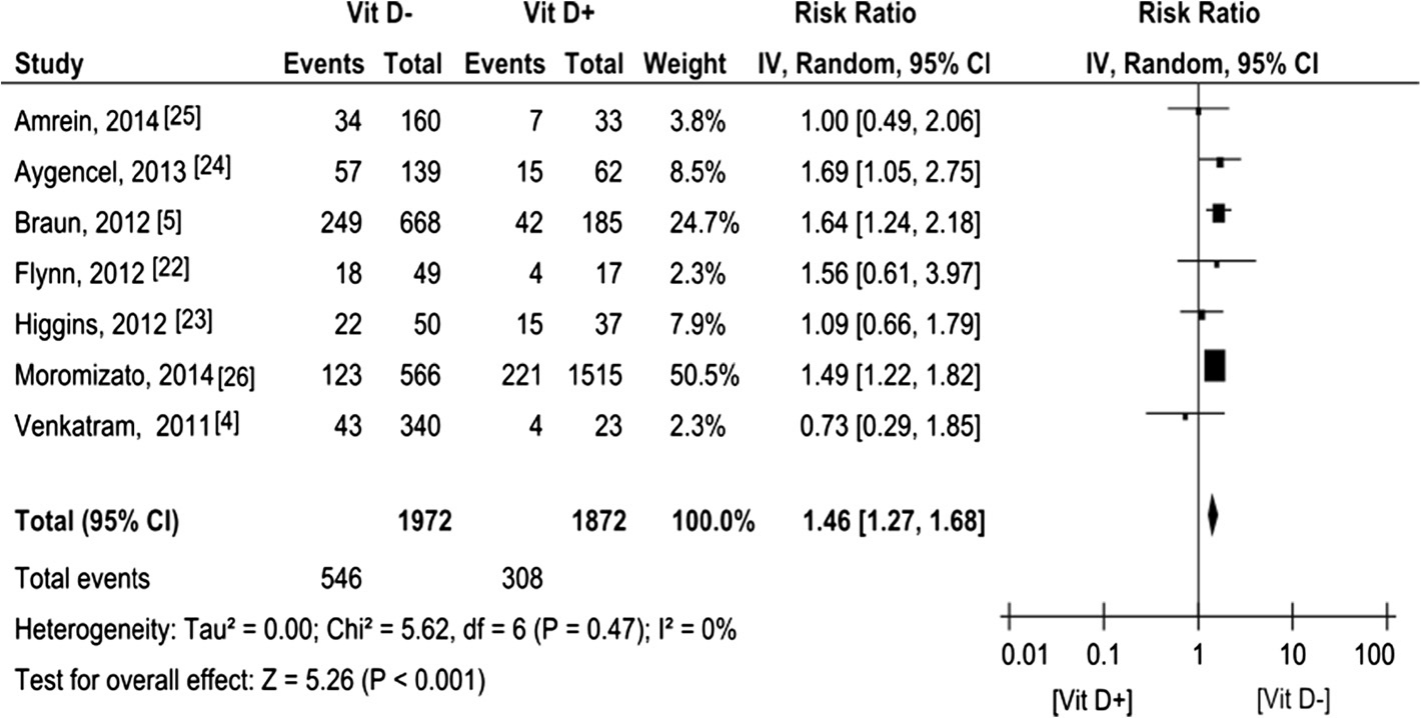
**Forest plot of studies comparing deficient vitamin D levels with sufficient vitamin D levels on sepsis.** CI, confidence interval; IV, inverse variance; Vit D-, deficient vitamin D level; Vit D+, sufficient vitamin D level.

The RRs in seven [4,5,22-26] of the fourteen studies were manually calculated, involving 3,844 patients (Figure 2). The pooled RR was 1.46 (95% CI 1.27 to 1.68).

### 25 (OH)-D levels and mortality

**Figure 3:**
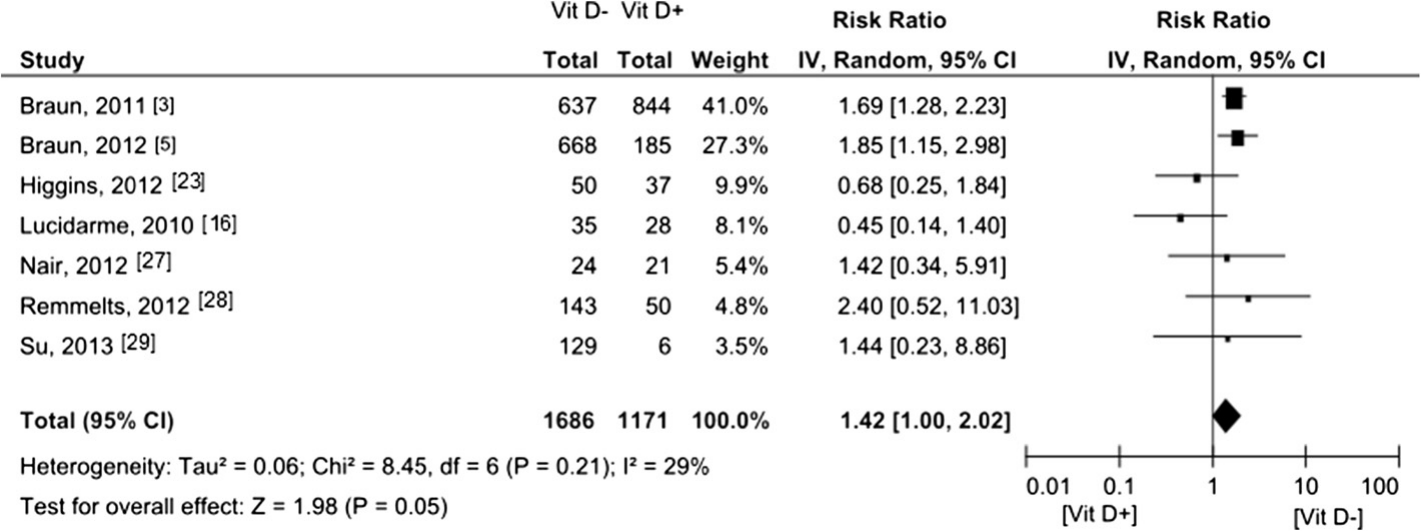
**Forest plot of studies comparing deficient vitamin D levels with sufficient vitamin D levels on 30 day-mortality (univariate analysis).** CI, confidence interval; IV, inverse variance; Vit D-, deficient vitamin D level; Vit D+, sufficient vitamin D level.

**Figure 4:**
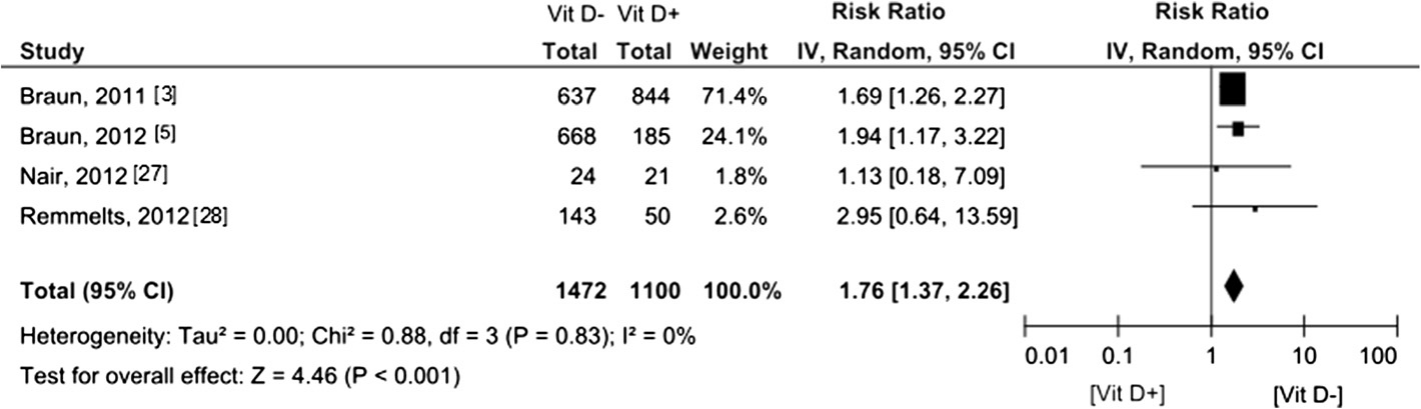
**Forest plot of studies comparing deficient vitamin D levels with sufficient vitamin D levels on 30 day-mortality (multivariate analysis).** CI, confidence interval; IV, inverse variance; Vit D-, deficient vitamin D level; Vit D+, sufficient vitamin D level.

**Figure 5:**
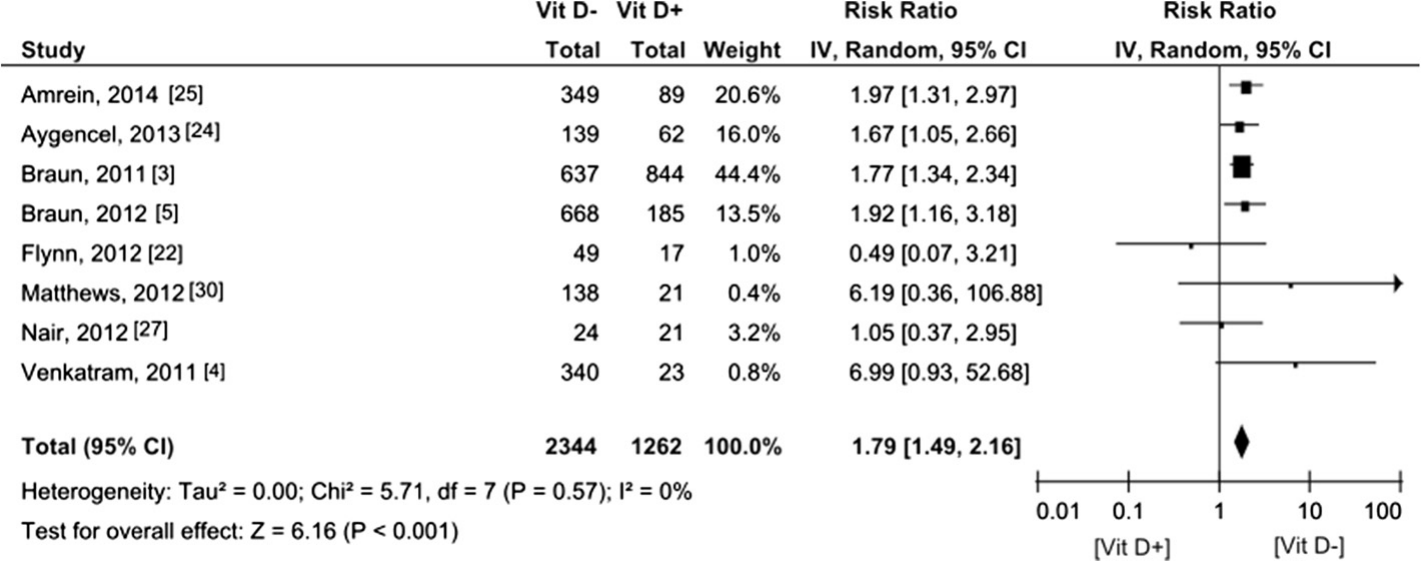
**Forest plot of studies comparing deficient vitamin D levels with sufficient vitamin D levels on in-hospital mortality.** CI, confidence interval; IV, inverse variance; Vit D-, deficient vitamin D level; Vit D+, sufficient vitamin D level.

Seven of the fourteen studies reported on 30-day mortality, with ORs either available in the articles converted to RRs [3,5] (n = 2) or manually calculated RRs [12,23,27-29] (n = 5), involving 2,857 patients (Figure 3). The pooled RR was 1.42 (95% CI 1.00 to 2.02). Two studies [3,5] used a multivariate model adjusted for age, gender, race, disease severity, season and ICU type. Remmelts *et al*. [28] adjusted for age and heart failure in the multivariate analysis and Nair *et al*. [27] for age and disease severity. The pooled subgroup analysis of adjusted data involving 2,572 patients demonstrated an increased RR for 30-day mortality associated with vitamin D deficiency by 1.76 (95% CI 1.37 to 2.26) (Figure 4). Eight [3-5,22,24,25,27,30] of the fourteen studies reported in-hospital mortality of 3,606 patients (Figure 5). The pooled RR was 1.79 (95% CI 1.49 to 2.16).

### Subgroup analyses per outcome based on study design

The effect on outcome in prospective study data is lower than in retrospective data see S10 in Additional file 1, but there were less prospective data.

## Discussion

This study represents the first comprehensive systematic review and meta-analysis focused on studies in which the effects of vitamin D deficiency in critically ill patients on occurrence of infection, sepsis and mortality rates are described. These results show that vitamin D deficiency (<50 nmol/L) is associated with increase in infection rate, sepsis, 30-day mortality and in-hospital mortality in adult critically ill patients, worldwide.

The association between vitamin D status and immunity has been already supported by a number of studies [31-33]. However, results in healthy volunteers exposed to experimental human endotoxaemia suggest a lack of this association between vitamin D and inflammatory cytokine levels [34]. Therefore, it is suggested that the differences in the ability to produce vitamin D, may contribute to a difference in the susceptibility to microbial infection. Our study adds to the discussion on the association by an objectively derived pooled risk. The relation between 25 (OH)-D levels and sepsis has been described previously [8]. Vitamin D-deficient patients are at higher risk for blood culture positivity, which may contribute to higher sepsis rates [3]. However, Cecchi *et al*. found no clear relationship on outcome between lower vitamin D levels in septic patients when compared with a matched cohort [16]. The most recent study by Moromizato *et al*. included in our analysis specifically showed a threshold of 25 (OH)-D less than or equal to 40 nmol/L to be associated with sepsis [26]. Thus our results are in agreement with the hypothesis that vitamin D deficiency is a contributor to sepsis.

Some published studies [17,35] suggested an association between vitamin D deficiency and mortality in critically ill patients. In the study by Van den Berghe *et al*. both 25 (OH)-D and 1,25 (OH)-D levels were lower among non-survivors in critically ill patients [17]. Matthews *et al*. noted in their surgical ICU cohort that most deaths occurred at vitamin D levels less than 32 nmol/L and that no deaths occurred at levels higher than 65 nmol/L [30]. The CopD study done by Durup *et al*. reported a reversed J-shape relation between 25 (OH)-D and all-cause mortality, suggesting that too much and too little are deleterious. A serum 25 (OH)-D of 50 to 60 nmol/L was associated with the lowest mortality risk [36]. The results of this meta-analysis suggest that vitamin D levels below 50 nmol/L, increase 30-day mortality and in-hospital mortality with 76% and 79% respectively. To date, only four randomised trials in adult critically ill patients have been published, which were designed to study normalisation of vitamin D levels and its possible adverse effects such as hypercalcaemia and hypercalciuria [17-20]. These studies were not sufficiently powered to investigate the effects of vitamin D normalisation and potential benefits on hard outcomes such as incidence of severe infections and/or ICU mortality (S11 Table 3 in Additional file 1). The recently published *Lancet* review supports the relation between 25 (OH)-D deficiency and all-cause mortality in observational studies [9]. The discrepancy with the interventional studies could be due to underpowered numbers, low dosages or short duration of supplementation. The role for supplementation is unclear and appropriate dose-response studies with 1,25 and 25 (OH)-D must be done. Therefore the authors’ conclusion about low vitamin D as merely a marker of disease has to be confirmed in prospective interventional studies.

Data from biochemical and molecular studies indicate that vitamin D, in particular its active form 1,25 (OH)-D, has a much wider role than only the maintenance of calcium homeostasis and bone health. Sufficiency of vitamin D activity can thus also be defined by sufficient autocrine and paracrine production of 1,25 (OH)-D at serum 25 (OH)-D levels of at least around 75 nmol/L [37,38]. This active form is responsible for most, if not all, of the biological and pleiotropic effects including antimicrobial actions and immunomodulatory effects of vitamin D [7,39]. The study by Zittermann *et al.* demonstrated the superiority of predicting mortality by 1,25 (OH)-D as compared to 25 (OH)-D, supporting the assumption that adequately circulating 1,25 (OH)-D levels may play a role for survival [40]. Marshall *et al.* emphasised measuring 1,25 (OH)-D instead of total 25 (OH)-D as well; they postulated that the disease processes regulate vitamin D metabolism so that the low 25 (OH)-D levels observed in disease may be merely a biomarker of disease severity [39]. Unfortunately, the observational articles meta-analysed in this manuscript do not consider 1,25 (OH)-D on outcomes.

Two studies [3,5] had a time lag between admission into the ICU and vitamin D blood sampling. To illustrate the importance between 25 (OH)-D time of measurement and admission, the authors conducted a sensitivity analysis considering patients with 25 (OH)-D drawn before or after 90 days prior to hospital admission. This sensitivity analysis showed that the association that was found between vitamin D on outcomes was not modified by time lag.

Our study has several limitations. First, we included both prospective and retrospective studies in this meta-analysis, which is a matter of debate. In retrospective studies the control for confounders is difficult. We have addressed this by adding a subgroup analyses per outcome based on design, see S10 in Additional file 1. However, in the prospective study data the effect on mortality is lower, whereas the sample size in the prospective studies may have been insufficient to show an association. The five retrospective papers [3-5,25,26] contain large sample sizes enabling multivariate analysis for mortality ruling out confounders such as age, gender, race, glomerular filtration rate (GFR), C-reactive protein (CRP), season, disease severity and so on. Second, the studies included are observational so that a causative link between hypovitaminosis D and outcomes cannot be established. Additionally, the variability in measured 25 (OH)-D levels is probably multifactorial. It is possible that a random single 25 (OH)-D measurement in ICU patients does not appropriately reflect the vitamin D status [41]. Furthermore, alterations in vitamin D binding protein, fluid shifts [42] and assay variability with coefficients of variation ranging from 6% to 13% [43] may limit applicability of single measurements on outcome prediction used in most of the included studies. The different cutoff levels used by different studies are based on study endpoints (for example, fracture or osteoporosis) done in the general population. The applicability of these cutoff levels in the critically ill is unclear, especially because cutoff values between bone- specific and pleiotropic endpoints are different. There is heterogeneity in the definitions of infection and sepsis used in the included studies, sepsis was defined varying from positive blood cultures [3] to SIRS criteria together with a source of infection [23] but the I^2^ test did not show heterogeneity. We only found some heterogeneity (*P* = 0.08) in the forest plot combining studies with infection as outcome.

## Conclusions

In conclusion, this is the first meta-analysis suggesting an association between vitamin D deficiency and infection and mortality in the critically ill. This information may help to design placebo-controlled randomised clinical trials on vitamin D supplementation in preventing severe infections and death in the ICU.

## Key messages

- 25 (OH)-D deficiency is highly prevalent across intensive care population worldwide.
- In critically ill patients, 25 (OH)-D deficiency is associated with mortality.
- 25 (OH)-D deficiency may be a risk factor for infections and sepsis.
- We support the need for adequately powered prospective, dose-response trials to evaluate the effect of vitamin D substitution on infection rates, sepsis and mortality in the critically ill.

## Additional file

Additional file 1: S1 Table 1. Results search strategy. **S2 Table 2.** Summary of characteristics of the included studies in the meta-analysis. **S3.** Funnel plot of studies comparing low vitamin D and normal vitamin D level on the occurrence of infections. **S4.** Funnel plot of studies comparing low vitamin D and normal vitamin D on the occurrence of sepsis. **S5.** Funnel plot of studies comparing low vitamin D and normal vitamin D on the occurrence of 30-day mortality (univariate). **S6.** Funnel plot of studies comparing low vitamin D and normal vitamin D on the occurrence of 30-day mortality (multivariate). **S7.** Funnel plot of studies comparing low vitamin D and normal vitamin D on the occurrence of in-hospital mortality. **S8.** Newcastle-Ottawa scale. **S9 Figure 1.** Flow diagram for the selection of studies evaluating the effect of vitamin D in critically ill patients. **S10.** Subgroup analyses per outcome based on study design. **S11 Table 3.** Summary of vitamin D interventional trials in the ICU.

## Abbreviations

1,25 (OH)-D: 1,25-dihydroxyvitamin D_3_
25 (OH)-D: 25-hydroxyvitamin D_3_
CI: confidence interval
CRP: C-reactive protein
GFR: glomerular filtration rate
ICU: intensive care unit
IV: inverse variance
MeSh: Medical Subject Heading
MOOSE: Meta-Analysis Of Observational Studies in Epidemiology guidelines
OR: odds ratio
PRISMA: Preferred Reporting Items for Systematic reviews and Meta-Analyses
PTH: parathyroid hormone
RR: risk ratio
SIRS: systemic inflammatory response syndrome
