# Supplementary material for: Vitamin D deficiency as a risk factor for infection, sepsis and mortality in the critically ill: systematic review and meta-analysis
Source: Crit Care. 2014 Dec 5;18(6):660. doi: 10.1186/s13054-014-0660-4 (PMC4277653; doi:10.1186/s13054-014-0660-4)

Additional file

Legend

S1 Table 1: Results search strategy.

S2 Table 2: Summary of characteristics of the included studies in the meta-analysis.

S3 Funnel plot of studies comparing low vitamin D and normal vitamin D level on the

occurrence of infections.

S4 Funnel plot of studies comparing low vitamin D and normal vitamin D on the occurrence

of sepsis.

S5 Funnel plot of studies comparing low vitamin D and normal vitamin D on the occurrence

of 30-day mortality (univariate).

S6 Funnel plot of studies comparing low vitamin D and normal vitamin D on the occurrence

of 30-day mortality (multivariate).

S7 Funnel plot of studies comparing low vitamin D and normal vitamin D on the occurrence

of in-hospital mortality.

S8 Newcastle-Ottawa-scale.

S9 Figure 1: Flow diagram for the selection of studies evaluating the effect of vitamin D in

critically ill patients.

S10 Subgroup analyses per outcome based on study design.

S11 Table 3: Summary of vitamin D interventional trials on the ICU.

**S1 Table 1: Results search strategy**

| Embase | 371 | 359 |
| --- | --- | --- |
| Medline in OvidSP | 112 | 12 |
| Cochrane central | 9 | 3 |
| Pubmed as supplied by publisher | 7 | 7 |
| Total | 500^a^ | 381^b^ |

a: before deduplication, b: after deduplication

Embase 371

('Vitamin D'/exp OR 'vitamin D deficiency'/de OR ('vitamin d' OR cholecalciferol* OR ergocalciferol* OR 'Hydroxyvitamin d' OR Hydroxycholecalciferol* OR 'Dihydroxyvitamin d' OR Dihydroxycholecalciferol* OR Calcifediol* OR Calcitriol*):ab,ti) AND ('intensive care'/de OR 'intensive care unit'/de OR 'intensive care nursing'/de OR 'critically ill patient'/de OR (((intens* OR critical*) NEAR/3 care*) OR (critical* NEAR/3 ill*)):ab,ti)

Medline in OvidSP 112

(exp "Vitamin D"/ OR "Vitamin D Deficiency"/ OR ("vitamin d" OR cholecalciferol* OR ergocalciferol* OR "Hydroxyvitamin d" OR Hydroxycholecalciferol* OR "Dihydroxyvitamin d" OR Dihydroxycholecalciferol* OR Calcifediol* OR Calcitriol*).ab,ti.) AND (exp "critical care"/ OR exp "intensive care units"/ OR "Critical Illness"/ OR (((intens* OR critical*) ADJ3 care*) OR (critical* ADJ3 ill*)).ab,ti.)

Cochrane central 9

(('vitamin d' OR cholecalciferol* OR ergocalciferol* OR 'Hydroxyvitamin d' OR Hydroxycholecalciferol* OR 'Dihydroxyvitamin d' OR Dihydroxycholecalciferol* OR Calcifediol* OR Calcitriol*):ab,ti) AND ((((intens* OR critical*) NEAR/3 care*) OR (critical* NEAR/3 ill*)):ab,ti)

Pubmed as supplied by publisher 7

(vitamin D*[tiab] OR Cholecalciferol*[tiab] OR Ergocalciferol*[tiab] OR Hydroxyvitamin d*[tiab] OR Hydroxycholecalciferol*[tiab] OR Dihydroxyvitamin d*[tiab] OR Dihydroxycholecalciferol*[tiab] OR Calcifediol*[tiab] OR Calcitriol*[tiab]) AND (intensive care*[tiab] OR critical care*[tiab] OR critical ill*[tiab] OR critically ill*[tiab]) AND publisher[sb]

**S2 Table 2: Summary of characteristics of the studies included in the meta-analysis.**

| **Author** | **Cutoffs of**  **25 (OH)-D** | **Definition of sepsis** | **Definition of infection** | **Study period** |
| --- | --- | --- | --- | --- |
| Amrein,2014^25^ | < 50 nmol/l  50-75 nmol/l  > 75 nmol/l | Positive blood culture | N/A | Between September 2008 and May 2010 |
| Arnson,2012^21^ | ≤ 50 nmol/l  > 50 nmol/l  Time samples taken: Within 24 hours after ICU admission | N/A | Respiratory, abdominal or other infection at ICU admission | Between  December 2008 and June 2009 |
| Aygencel,2013^24^ | < 50 nmol/l  ≥ 50 nmol/l  Time samples taken:  Within 24 hours after ICU admission | N/A | Nosocomial infections | Between October 2009 and March 2011 |
| Braun,2011^3^  2012^5^ | ≤ 37 nmol/l  40-75 nmol/l  ≥ 75 nmol/l  Time samples taken: 7 until 365 days before ICU admission ^6^.  7 days before until 7 days after ICU admission ^21^. | ICD-9-CM codes:  038.0-038.9, 020.0, 790.7, 117.9, 112.5, 112.81 | Positive blood culture | Between 1998-2009 |
| Flynn,2012^22^ | ˂ 50 nmol/l  ≥ 50 nmol/l  Time samples taken: On admission and every 7 days | N/A | Overall infections including pneumonia, intra-abdominal, urinary tract, skin-soft tissue | Between January 2010 and February 2011 |
| Higgins,2012^23^ | ≤ 30 nmol/l  > 30-≤60 nmol/l  > 60 nmol/l  Time samples taken: Within 24 hours after ICU admission and daily for 10 days. | At any time during stay patients met SIRS criteria and had a source of infection. | ICU-acquired infection; present after 48 hours of ICU admission.  Suspected infection; by the presence of new positive culture or initiation of new AB after 48 hours of ICU admission | Between October 2002 to October 2003  Summer was defined as; admission between 21 June and 22 September |
| Lucidarme,2010^16^ | ≤ 15 nmol/l  > 15-≤ 30 nmol/l  > 30-≤ 60 nmol/l  > 60 nmol/l  Time samples taken:  On admission | N/A | N/A | Spring-Summer 2009 |
| Matthews,2012^30^ | ≥ 10- ≤32 nmol/l  33-65 nmol/l  66-99 nmol/l  ≥ 100- ≤175 nmol/l  Time samples taken: Within 24 hours after ICU admission | N/A | N/A | Between August 2009 and January 2011 |
| Moromizato,2014^26^ | ≤ 37 nmol/l  40-75 nmol/l  ≥ 75 nmol/l | ICD-9-CM codes:  038.0-038.9, 020.0, 790.7, 117.9, 112.5, 112.81, 3 days prior to critical care initiation to 7 days after critical care initiation. | N/A | Between 1998-2011 |
| Nair,2012^27^ | < 25 nmol/l  25-50 nmol/l  > 50 nmol/l  Time samples taken:  Within 24 hours after admission and in the morning on day 3 and 7 or at ICU discharge | N/A | N/A | Between July 2010 and February 2011 |
| Remmelts,2012^28^  Su,2013^29^  Venkatram,2011^4^ | < 50 nmol/l  50-75 nmol/l  > 75 nmol/l  < 12,5 nmol/l  12,5-25 nmol/l  > 25-≤37,5  > 37,5-≤50  > 50 nmol/l  Time samples taken:  Within 24 hours after ICU admission  < 50 nmol/l  ≥ 50-75 nmol/l  ≥ 75 nmol/l  Time samples taken:  Within 24 hours after ICU admission | N/A  N/A  N/A | Community acquired pneumonia  N/A  N/A | 1 Year  Between October and December 2011  Between October 2009 and February 2010 |

N/A: not available; ICU: intensive care unit; SIRS: systemic inflammatory response syndrome; AB: antibiotic

**S3 Funnel plot of studies comparing low vitamin D and normal vitamin D level on the**

**occurrence of infections.**

**
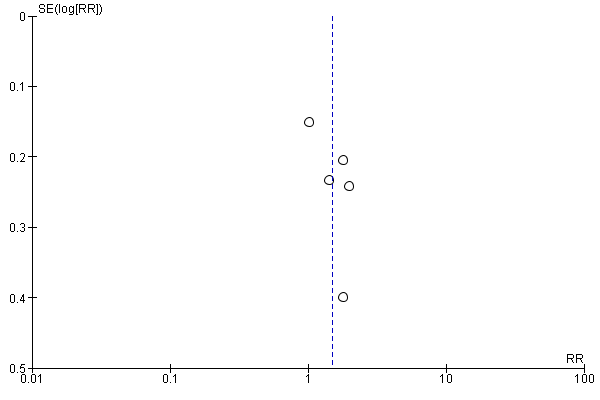
**

RR; relative risk, SE; standard error.

**S4 Funnel plot of studies comparing low vitamin D and normal vitamin D on the occurrence**

**of sepsis.**

**
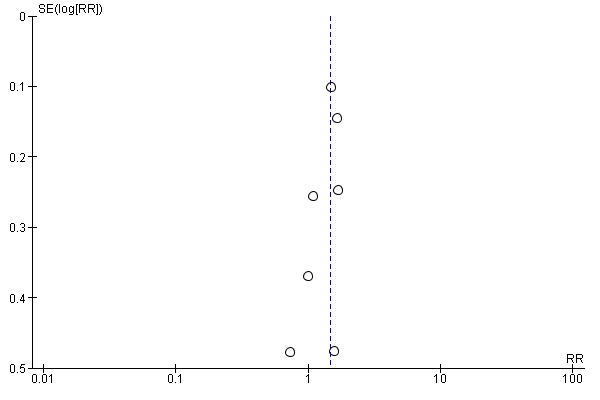
**

RR; relative risk, SE; standard error.

**S5 Funnel plot of studies comparing low vitamin D and normal vitamin D on the occurrence**

**of 30-days mortality (univariate).**

**
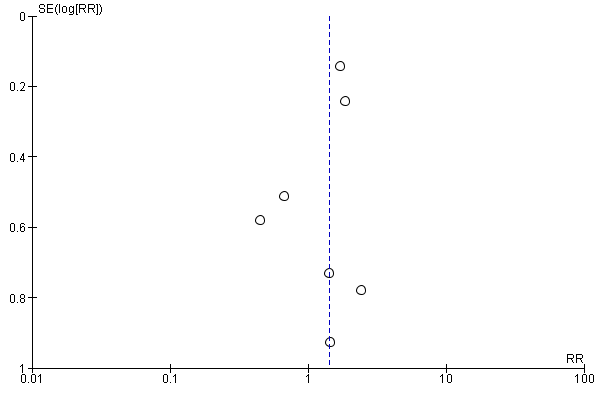
**

RR; relative risk, SE; standard error.

**S6 Funnel plot of studies comparing low vitamin D and normal vitamin D on the occurrence**

**of 30-days mortality (multivariate).**

**
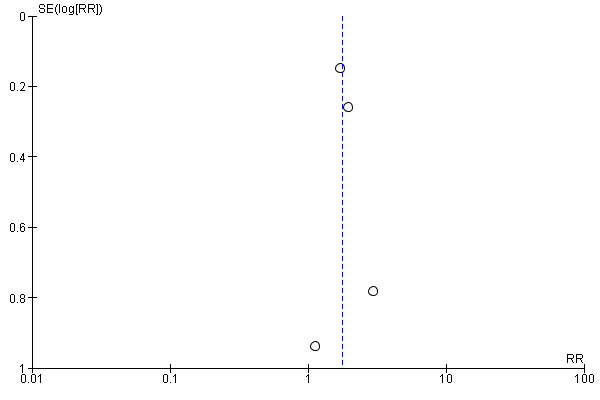
**

RR; relative risk, SE; standard error.

**S7 Funnel plot of studies comparing low vitamin D and normal vitamin D on the occurrence**

**of in-hospital mortality.**

**
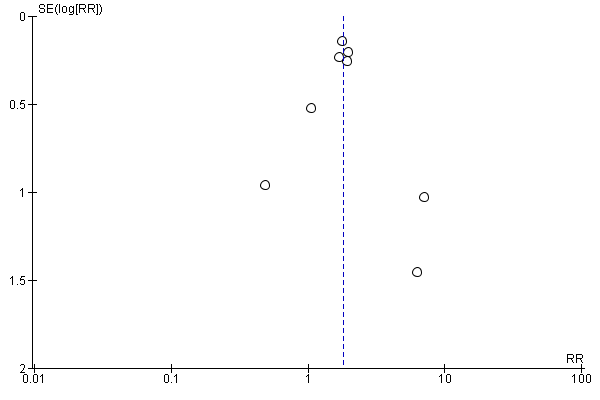
**

RR; relative risk, SE; standard error.

**S8a Newcastle-Ottawa-scale for cohort studies data abstraction form**


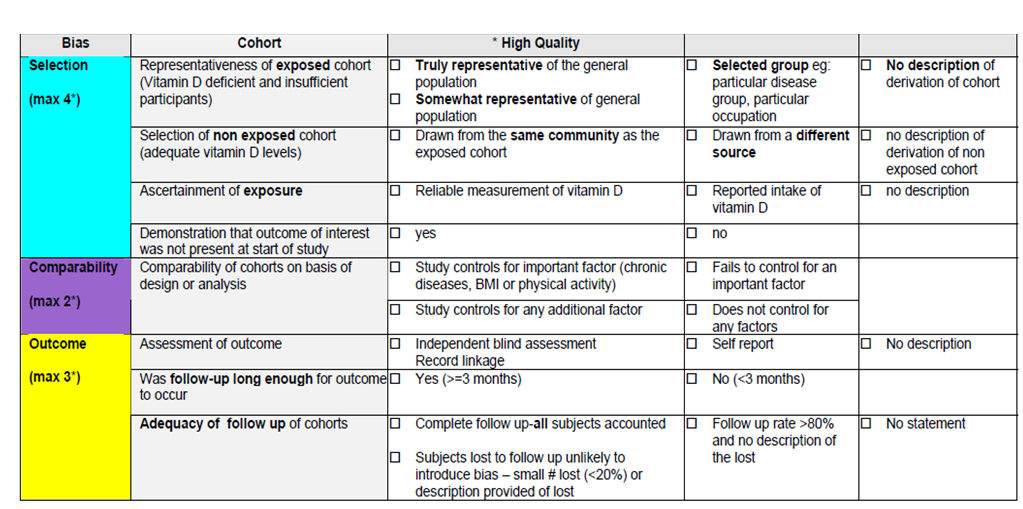


**S8b Risk of bias summary for cohort studies: review authors judgments about each risk of bias item for each included study using the Newcastle-Ottawa-Scale.**

|  | Representativeness of exposed cohort (vitamin D deficient patients) | Selection of non exposed cohort (adequate vitamin D levels) | Ascertainment of exposure | Demonstration that outcome of interest was not present at start of study | Comparability of cohorts on basis of design or analysis (max 2 points) | Assessment of outcome | Was follow-up long enough for outcome to occur | Adequacy of follow-up of cohorts | Total points/9 |
| --- | --- | --- | --- | --- | --- | --- | --- | --- | --- |
| Amrein^25^ | 1 | 1 | 1 | 1 | 1 | 1 | 1 | 0 | 7/9 |
| Arnson^21^ | 1 | 1 | 1 | 1 | 0 | 1 | 1 | 0 | 6/9 |
| Aygencel^24^ | 1 | 1 | 1 | 1 | 0 | 0 | 0 | 0 | 4/9 |
| Braun,2012^5^ | 1 | 1 | 1 | 1 | 2 | 1 | 1 | 0 | 8/9 |
| Braun,2011^3^ | 1 | 1 | 1 | 1 | 2 | 1 | 1 | 0 | 8/9 |
| Flynn^22^ | 0 | 1 | 0 | 0 | 0 | 0 | 1 | 0 | 2/9 |
| Higgins^23^ | 1 | 1 | 1 | 1 | 1 | 1 | 1 | 0 | 7/9 |
| Lucidarme^16^ | 1 | 1 | 1 | 1 | 1 | 0 | 0 | 0 | 5/9 |
| Matthews^30^ | 1 | 1 | 0 | 1 | 0 | 0 | 0 | 0 | 3/9 |
| Moromizato^26^ | 1 | 1 | 1 | 1 | 2 | 1 | 1 | 0 | 8/9 |
| Remmelts^28^ | 1 | 1 | 0 | 1 | 2 | 1 | 1 | 0 | 7/9 |
| Su^29^ | 1 | 1 | 1 | 1 | 1 | 0 | 1 | 0 | 6/9 |
| Venkatram^4^ | 1 | 1 | 1 | 0 | 0 | 0 | 1 | 0 | 4/9 |

| 9-8 pts | Low risk of bias |
| --- | --- |
| 7-6 pts | Moderate risk of bias |
| <6 pts | High risk of bias |

**S9 Figure 1: Flow diagram for the selection of studies evaluating the effect of vitamin D in critically ill patients.**

Search results March 2014: (n=381)

Embase (n=359)

Medline (n=12)

Cochrane central (n=3)

Pubmed as supplied by publisher (n= 7)

studies excluded after title and abstract screening by inclusion criteria, described in methods. (n=358)

irrelevant/other subject (n=229)

vitamin D metabolism (n=45)

study design or n<20 (n=26)

review (n=7)

pediatric (n=46)

animal studies (n=5)

Paediatric (n=46), Animals (n=5)

studies retrieved for detailed evaluation (n=23)

studies excluded

duplicate (n=1)

studies to be included in the systematic review (n=22)

studies excluded

unable to pool results in meta-analysis (n=4)

intervention trials (n=4)

studies to be included in the meta-analysis (n=14)

reporting on:

infections (n=5)

sepsis (n=7)

30-day mortality (n=7 )

in-hospital mortality (n=8)

**S10 Subgroup analyses per outcome based on study design.**

| **Outcome** | **n** | **ref** | **RR, [95% CI], p** |
| --- | --- | --- | --- |
| **Infection**  *Prospective studies*  Arnson, 2012  Aygencel, 2013  Flynn, 2012  Higgins, 2012  *Retrospective studies*  Braun, 2011 | 4  1 | 21  24  22  23  5 | 1.42, [1.01, 2.00], 0.04  1.80, [1.20, 2.70], <0.001 |
| **Sepsis**  *Prospective studies*  Aygencel, 2013  Flynn, 2012  Higgins, 2012  *Retrospective studies*  Amrein, 2014  Braun, 2012  Moromizato, 2014  Venkatram, 2011 | 3    4 | 24  22  23  25  5  26  4 | 1.39, [1.00, 1.92], 0.05  1.45, [1.18, 1.78], <0.001 |
| **30-day mortality (univariate)**  *Prospective studies*  Higgins, 2012  Lucidarme, 2010  Nair, 2012  Remmelts, 2012  Su, 2013  *Retrospective studies*  Braun, 2011  Braun, 2012 | 5    2 | 23  16  27  28  29  5  3 | 0.89, [0.50, 1.58], 0.69  1.73, [1.36, 2.20], <0.001 |
| **30-day mortality (multivariate)**  *Prospective studies*  Nair, 2012  Remmelts, 2012  *Retrospective studies*  Braun, 2011  Braun, 2012 | 2  2 | 27  28  5  3 | 1.99, [0.62, 6.45], 0.25  1.75, [1.36, 2.26], <0.001 |
| **In-hospital mortality**  *Prospective studies*  Aygencel, 2014  Flynn, 2012  Matthews, 2012  Nair, 2012  *Retrospective studies*  Amrein, 2014  Braun, 2011  Braun, 2012  Venkatram, 2011 | 4  4 | 24  22  30  27  25  5  3  4 | 1.51, [1.00, 2.27], 0.05  1.87, [1.52, 2.31], <0.001 |

**S11 Table 3. Overview of Intervention trials on the ICU**

| Author | Year of Publication | Study  population | Study design | No. of patients | Endpoints |
| --- | --- | --- | --- | --- | --- |
| Amrein ^19^  Mata- Garandos^18^  Van den Berghe ^17^  Vargas-Vasserot ^20^ | 2011  2010  2003  2011 | medical-,  ICU  ICU  Surgical-ICU  ICU | prospective double-blind  randomized placebo-controlled  randomized controlled  randomized, controlled  randomized, controlled | 25  placebo  n=13  13.5 mg  cholecalciferol PO  n= 12  33  controls  n= 11  1,5 mg cholecalciferol PO  n= 11  2 mcg  calcitriol i.v  n= 10  22  low dose  5 mcg cholecalciferol i.v  n=10  high dose  12,5 mcg  cholecalciferol i.v  n=12  23  controls  n=12  1,5 mg cholecalciferol  n=11 | normalization of 25 (OH)-D, mechanical ventilation, vasopressor therapy, hospital stay, ICU stay, hospital mortality  normalization of 25 (OH)-D, 1,25 (OH)-D,  24,25 (OH)-D  normalization of 25 (OH)-D, 1,25 (OH)-D, VDBP, PTH, iCa,  IL-6, IL-1, CRP, markers of bone formation  normalization of 25 (OH)-D, 1,25 (OH)-D, 24,25 (OH)-D,  cathelicidin (LL-37) |


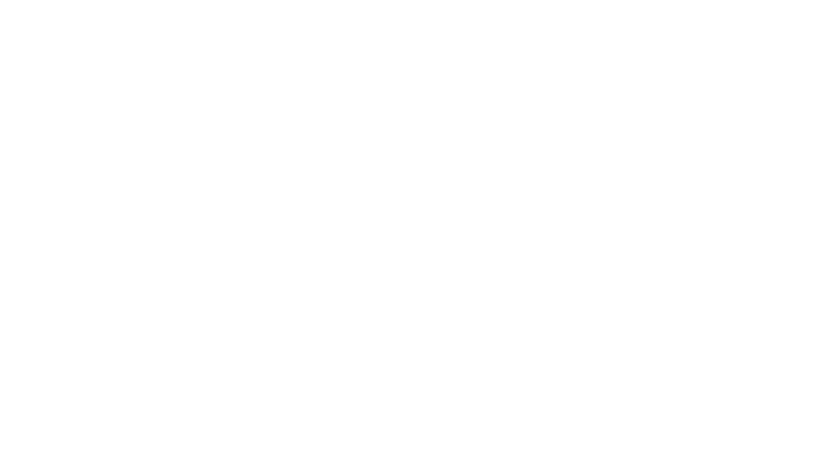

Supplement: Additional file 1: S1 Table 1. — Results search strategy. S2 Table 2. Summary of characteristics of the included studies in the meta-analysis. S3. Funnel plot of studies comparing low vitamin D and normal vitamin D level on the occurrence of infections. S4. Funnel plot of studies comparing low vitamin D and normal vitamin D on the occurrence of sepsis. S5. Funnel plot of studies comparing low vitamin D and normal vitamin D on the occurrence of 30-day mortality (univariate). S6. Funnel plot of studies comparing low vitamin D and normal vitamin D on the occurrence of 30-day mortality (multivariate). S7. Funnel plot of studies comparing low vitamin D and normal vitamin D on the occurrence of in-hospital mortality. S8. Newcastle-Ottawa scale. S9 Figure 1. Flow diagram for the selection of studies evaluating the effect of vitamin D in critically ill patients. S10. Subgroup analyses per outcome based on study design. S11 Table 3. Summary of vitamin D interventional trials in the ICU. [file 13054_2014_660_MOESM1_ESM.docx]
